# Senescence in HBV-, HCV- and NAFLD- Mediated Hepatocellular Carcinoma and Senotherapeutics: Current Evidence and Future Perspective

**DOI:** 10.3390/cancers13184732

**Published:** 2021-09-21

**Authors:** Vassilis G. Giannakoulis, Peter Dubovan, Eleni Papoutsi, Agapi Kataki, John Koskinas

**Affiliations:** 1 First Department of Propaedeutic Surgery, “Hippokratio” General Hospital, Vasilissis Sofias 114, 11 527 Athens, Greece; helenapapoutsi@gmail.com (E.P.); akataki@med.uoa.gr (A.K.); 2 Medical School, National and Kapodistrian University of Athens, Mikras Assias 75, 11 527 Athens, Greece; koskinasj@yahoo.gr; 3 Department of Surgical Oncology, National Cancer Institute in Bratislava, Slovak Medical University, Klenova 1, 833 10 Bratislava, Slovakia; peter.dubovan@nou.sk; 4 Biomedical Research Center SAS, Cancer Research Institute, Dúbravská Cesta 9, 845 05 Bratislava, Slovakia; 5 Second Department of Internal Medicine, “Hippokratio” General Hospital, Vasilissis Sofias 114, 11 527 Athens, Greece

**Keywords:** cell senescence, hepatocellular cancer, hepatitis, non-alcoholic fatty liver disease, senotherapeutics, senescence-modulating agents, senomorphics, senoptotics

## Abstract

**Simple Summary:**

Recent scientific discoveries identify cell senescence as pivotal in hepatocellular cancer (HCC) biology. Specifically, hepatitis B virus (HBV), hepatitis C virus (HCV) and non-alcoholic fatty liver disease (NAFLD) are major risk factors for HCC occurrence and it seems that cell senescence serves as a mediator. Furthermore, senescence is also implicated in HCC therapy resistance. Therefore, understanding and harnessing senescence (via senotherapeutics) seems highly important towards the discovery of new preventative and treatment strategies. Herein, we review the role of cell senescence in HBV-, HCV- and NAFLD- mediated HCC, and also explore the possible place of senotherapeutics in the management of HCC. By shining the spotlight on senescence-mediated HCC, we aim to inspire future research towards this rapidly evolving and highly promising field.

**Abstract:**

Cell senescence constitutes a physiological process that serves as protection from malignant transformation of cells. However, recent scientific discoveries also identify cell senescence as pivotal in hepatocellular cancer (HCC) biology. The review herein aimed to accumulate evidence on senescence as a mediator of HCC occurrence in hepatitis B (HBV), C (HCV) virus infections, and non-alcoholic fatty liver disease (NAFLD). In HBV infection, the carcinogenic HBV X protein frequently mutates during chronic infection, and subsequently exhibits different effects on senescence. In HCV infection, senescent non-functional T-cells do not effectively clear pre-malignant hepatocytes. Furthermore, the HCV Core protein inhibits the occurrence of normal stress-induced hepatocyte senescence, allowing damaged cells to maintain their proliferative potential. In NAFLD-mediated HCC, current data point towards the gut microbiome and hepatic stellate cell senescence. Additionally, senescence contributes in the development of resistance in targeted therapies, such as sorafenib. Finally, the promising role of senotherapeutics in HCC was also explored. Overall, although we may still be at a primitive stage in fully unraveling the role of senescence in cancer, it seems that understanding and harnessing senescence may have the potential to revolutionize the way we treat hepatocellular cancer.

## 1. Introduction

When Hayflick and Moorhead first described cell senescence in 1961 [1], their findings were received with skepticism. Their manuscript was initially rejected [2] and decades passed before the wide acceptance of this concept [3]. Today, the scientific community acknowledges cell senescence as pivotal in cancer biology and aging research, whereas an emerging new field, known as senotherapeutics, aims to create senescence-modulating agents that affect the course of age-related diseases, such as cancer [4].

Recent scientific advances indicate the importance of senescence in hepatocellular carcinoma (HCC) [5,6], which represents a major cause of mortality worldwide, with a substantial societal and economic burden. Hepatitis B virus (HBV), hepatitis C virus (HCV) and non-alcoholic fatty liver disease (NAFLD), are major risk factors for HCC development. Provided that the tumor is detected at an early stage, resection, ablation or liver transplantation are the potentially curative options [7]. Nonetheless, patients are often diagnosed at an advanced stage, which requires systemic treatment. In this patient subgroup, the concept of targeted therapies has stimulated substantial research, with promising drugs being introduced in the period between 2017–2020 [8]. Sorafenib, a multiple tyrosine kinase inhibitor (TKI), has been for almost a decade the standard of care treatment for HCC, while more recently other TKIs have shown efficacy as first or second line treatment. Currently, immunotherapy, the combination of atezolizumab plus bevacizumab, has been approved as first line treatment for HCC [9,10]. Even though sorafenib has been proven to exert benefits, its overall efficacy is low [11,12]. Genetic heterogeneity possibly contributes to primary resistance of HCC cells (i.e., resistance without prior exposure to sorafenib), which is frequently reported [13]. Furthermore, patients initially responsive to sorafenib treatment will acquire resistance to therapy, also known as acquired resistance [14]. Thus, despite promising advances, further research is needed on the underlying mechanisms and molecular signature of HCC, which will subsequently pave the way for personalized treatment strategies.

Herein, we aimed to review the role of cell senescence in HBV-, HCV- and NAFLD- mediated HCC, as well as to explore the possible place of senotherapeutics in the management of HCC.

## 2. Liver Cell Senescence: Definition and Characteristics

### 2.1. Definition

Cell senescence is a cellular state that entails: (a) cell-cycle arrest, (b) macromolecular damage, (c) senescence-associated secretory phenotype (SASP), and (d) deranged metabolic profile. Thus, it is often considered as a grey zone between cellular death and survival [15]. In the first study on senescence, Hayflick described a state of aging on a cellular level, in which cells, after about 50 replications, enter senescence (a term inspired from the Latin word “senex”, meaning old) [1]. This finding was later explained through the telomere theory. According to this theory, cells enter natural cell-cycle arrest after a finite number (“Hayflick limit”) of cell replications (replicative senescence), to avoid genomic instability due to telomere shortening [16]. Consequently, in this context, senescence is a physiological process serving as protection from malignant transformation of aged cells.

Nonetheless, contemporary research has made clear that factors beyond normal cellular aging can serve as potential inducers of cell senescence. These are generally stress-related and include: genotoxic drugs (e.g., chemotherapy), irradiation, oncogene activation, cytokines (TGF-β), epigenetic modifiers (e.g., Curcumin), high-fat diets and ribosomal stress [15]. Oncogene-induced senescence (OIS) is possibly an important initial barrier against HCC occurrence [17]. OIS describes the “reflexive” induction of senescence in cells that receive excessive oncogenic stimuli, which subsequently protects against the uncontrolled proliferation of damaged cells [18].

As senescence mainly describes cell-cycle arrest of previously growth-capable cells, tissues with renewable properties have been reported to be more vulnerable [19]. Moreover, chronic inflammation has been strongly linked to cell senescence [20]. Thus, in accordance with the above-mentioned, senescence has been detected on liver tissues derived from individuals with HBV infection, HCV infection, NAFLD and HCC [21,22,23,24]. Notably, and contrary to the norm, hepatocytes and cholangiocytes from highly selected healthy liver tissue were found to be very durable to replicative senescence (i.e., aging), as they exhibit preserved telomere length over a wide range of ages [25]. This highlights the crucial role of stress (i.e., chronic liver disorders, injury and inflammation) as an instigator of senescence in the liver.

Finally, identifying senescent cells still poses a challenge, as they lack specific markers. It has been recommended that initial screening should be attempted via the detection of beta-galactosidase activity and/or lipofuscin accumulation [15]. Furthermore, many studies suggest the expression of cyclin-dependent kinase inhibitor CDKN1A (also known as p21^CIP^ or p21), CDKN2A (also known as p16^INK4A^ or p16) and CDKN2B (also known as p15^INK4B^ or p15) as strong additional identifiers of cell senescence [15,26,27,28,29,30].

### 2.2. The Janus Face of Senescence: Senescence-Associated Secretory Phenotype

Although senescence is considered as a natural protective mechanism against tumorigenesis and even more as a potential treatment strategy [31], it exhibits duality similar to the Roman god Janus [32] which means that under certain circumstances it can be carcinogenic via the senescence-associated secretory phenotype (SASP) [33]. The SASP is a pro-inflammatory response that is crucial in the immune-mediated clearance of senescent cells via phagocytosis [34]. In addition, it involves the secretion of various factors, such as interleukins (with the most prominent being IL-6), chemokines such as IL-8 (CXCL-8) and matrix metalloproteinases (MMP) [33]. All these factors affect the tumor microenvironment, which is defined as anything non-cancerous inside a tumor mass (including vessels, immune cells, secreted factors, extracellular matrix). Recently, the tumor microenvironment has been reported as an important driver of cancer biology facilitating cancer progression and metastasis [35,36]. Hence, the accumulation of senescent cells may significantly affect the tumor microenvironment via the SASP, and thus promote cancer progression.

More specifically, in the liver, it has been reported that senescent hepatic stellate cells release increased quantities of extracellular vesicles compared to non-senescent ones, which subsequently augments epidermal growth factor (EGF) secretion from the macrophages [37]. This may result in a more permissive tumor microenvironment for cell proliferation. In another study, SASP-mediated CCL2-CCR2 signaling promoted the growth of HCC via the attraction of immature myeloid cells, which inhibit natural killer cells. At the same time, the CCL2-CCR2 axis was also deemed important in the clearance of senescent cells, which further highlights the context-dependent role of SASP [38].

### 2.3. The Fate of Senescent Cells

**Figure 1:**
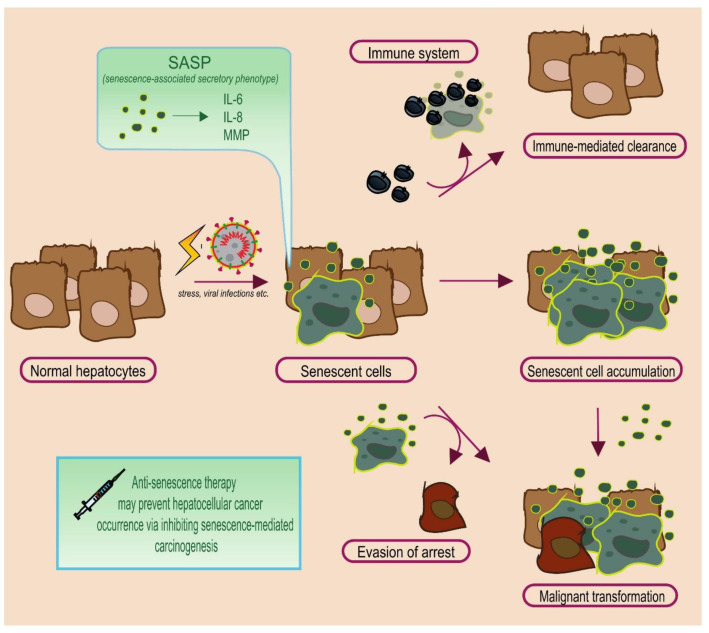
Emergence and fate of senescent hepatocytes. Initially, a stress factor induces cellular damage and triggers the formation of senescent cells. Scenario 1: Activation of the immune system via the senescence-associated secretory phenotype (SASP) and clearance. Scenario 2: Abnormal overt accumulation, SASP secretome activation and carcinogenesis. Scenario 3: Pre-malignant hepatocytes evade senescence arrest and gain proliferative potential, contributing to hepatocellular cancer occurrence. Administering anti-senescence agents may enhance senescent cell elimination and ameliorate their disastrous potential. Normal hepatocytes are presented in pale brown, malignant ones in dark brown, whereas senescent ones are presented in light blue with yellow outline.

On a favorable scenario, hepatic senescent cells are identified and cleared by the immune system [39,40]. In some instances, senescent cells return back to a normal state [41,42], although senescence is generally considered an irreversible state [15]. However, concerns are raised when senescent cells over-accumulate or when they abnormally manage to evade senescence. The former allows for a permissive tumor microenvironment, via the SASP [33]. The latter implies that damaged cells manage to evade a normal checkpoint of proliferative arrest, which may subsequently promote carcinogenesis and/or cancer progression [39,43]. Figure 1 depicts the possible pathways that senescent hepatocytes can follow.

## 3. Senescence in HBV-, HCV-, and NAFLD-Mediated HCC

The pathophysiological role of senescence in HBV-, HCV-, and NAFLD-mediated HCC will be analyzed in this section; key discoveries are summarized on Table 1.

### 3.1. Hepatitis B Virus (HBV): HBx Protein

The HBV x protein (HBx), a 17 kD transcriptional activator, has been implicated in both carcinogenesis [44,45], and senescence [46]. Intriguingly, during chronic infection, the HBV genome undergoes alterations, especially in the HBx gene, which results in 3′-truncated HBx sequences [47,48,49,50]. As a result, HBx C-terminal mutant proteins are produced, which have different, and sometimes, opposite properties regarding the induction of senescence [51]. For example, HBx C-terminal mutants have been found to induce senescence in primary MRC5 cells, malignant Huh7 cells, and SK-Hep1 cells, whereas they promoted proliferation in HepG2 malignant liver cells [51]. Notably HBx has also been shown to play a key role in the evasion of damaged cells from senescence [52,53].

HBx also promotes the expression of factors of the SASP phenotype by triggering MMP [54] and IL-6 expression [55]. In particular, pre-operative serum IL-6 levels have been proposed as a prognostic factor of HCC recurrence on a background of HBV infection [56]. Finally, the HBx protein has been implicated in telomere shortening [57], which leads to hepatocellular senescence and consequently the development of cirrhosis [58].

Overall, the accumulated data suggest a pivotal role of HBx in senescence-mediated HCC occurrence and progression. However, it seems that its effect on senescence may depend on the specific HBx isoform, as well as to cellular characteristics [51]. Considering that the HBx gene is one of the most frequently mutated genes during chronic HBV infection, an interesting topic of future studies would be to further explore how different isoforms of HBx enhance HCC occurrence and progression at different stages of chronic HBV infection.

### 3.2. Hepatitis C Virus (HCV): T-Cell Senescence and HCV Core Protein

Chronic hepatitis C and the associated liver inflammation accelerate the telomere-shortening process and, thus, constitute a state of replicative senescence that predisposes to HCC [59]. While chronic HBV infection can circumstantially cause HCC in the absence of cirrhosis [60], HCV-mediated HCC usually develops in the cirrhotic patient [61]. Moreover, it has been proposed that the level of cell senescence reflects the progression of liver fibrosis in patients with chronic hepatitis C [23]. Interestingly, senescent markers appear more evident in non-parenchymal fibrotic tissue, an observation attributed to increased proportion of intrahepatic senescent T-cells [23]. These senescent T-cells are non-functional, and their presence occupies vital liver space where active immune cells could be located [62]. The reduced activity of T-cells in HCV infection may predispose to HCC occurrence, as T-cells orchestrate the clearance of senescent pre-malignant hepatocytes [39].

Apart from precipitating T-cell senescence, studies on human liver cell lines suggested that HCV also suppresses premature hepatocellular senescence imposed by oxidative stress [63]. Oxidative stress constitutes an important pathophysiologic mechanism of cellular damage in chronic hepatitis C triggering DNA alterations and subsequently causing HCC [64]. Considering that the presence of oxidative stress also causes stress-induced premature senescence (SIPS), normal mechanisms of senescence are impaired so that hepatocytes exposed to oxidative stress maintain their proliferative potential. Indeed, the HCV Core protein overcomes premature senescence provoked by a reactive oxygen species inducer, H_2_O_2_, in human liver cells. The proposed mechanism is down-regulation of p16 via hypermethylation of its promoter [63,65].

### 3.3. Non-Alcoholic Fatty Liver Disease (NAFLD)

Cellular senescence seems to be implicated in NAFLD pathogenesis and progression according to both animal and human studies, and mediates obesity-related HCC [66,67,68,69]. NAFLD has been described as an “umbrella term” encompassing every liver disease presented with deposition of excess macro vesicular fat (>5% of the hepatocytes) in the absence of alcohol intake, drug exposure or relevant genetic disorders [70,71]. This abnormal liver state actually represents the hepatic manifestation of insulin resistance and is strongly associated with obesity [72], while current evidence suggests that it constitutes an independent risk factor for HCC, even in the absence of cirrhosis [73].

Hepatic stellate cells senescence induced by gut microbiota alterations in an obese context proposes a potential mechanism for development of HCC in NAFLD [74,75]. More specifically, a landmark study revealed that upregulation of deoxycholic acid (DCA) in obese mice, a gut bacterial metabolite that transfers via enterohepatic circulation, facilitates SASP phenotype in hepatic stellate cells and HCC development [74]. The role of SASP as a mediator of obesity-related HCC was ascertained by experiments showing that mice lacking IL-1b gene, a SASP upstream regulator, presented a mitigated size and number of HCC tumors. Notably, this observation was further supported by the fact that administration of vancomycin, an antibiotic diminishing gut microbiota in obese mice, blocked hepatic stellate cell senescence and HCC development [74]. Additionally, DNA microarray studies on a human hepatic stellate cells line strengthened the notion that DCA promotes senescence, and identified TGFβ and IL-8 as the key SASP factors promoting HCC migration and invasion [75,76]. Another study on mice revealed that lipoteichoic acid (LTA), an obesity-induced Gram-positive gut microbial component, also promotes hepatic stellate cells senescence. Hence, cooperatively with DCA, LTA, induces the expression of SASP factors and cyclooxygenase-2 (COX2) through Toll-like receptor 2 [69]. Moreover, prostaglandin E2, a lipid mediator generated by COX2, overproduced by senescent hepatic stellate cells suppresses antitumor immunoactivity and thus participates in HCC pathophysiology [69].

On the pathophysiological mechanism of hepatic stellate cell contribution in hepatocellular carcinoma, a recent study revealed that loss of the gluconeogenic enzyme FBP1 (fructose 1,6-bisphosphatase 1) promotes HCC development through crosstalk of senescent hepatic stellate cells and hepatocytes [77]. In the same study, it was observed that the senolytics Dasatinib + Quercetin or ABT-263 (Navitoclax) are effective against NAFLD-related HCC caused by the loss of FBP1 [77]. Overall, the current data point towards hepatic stellate cell senescence and alterations in the gut microbiome as contributing factors of senescence-mediated HCC in NAFLD.

Of note, the recently approved first-line immunotherapy seems to have an impaired (and even pro-tumorigenic) effect against non-viral causes of HCC, such as non-alcoholic steatohepatitis (NASH) [78]. The proposed mechanism behind this finding is that immunotherapy activates CD8^+^ T-cells, which in NAFLD are exhausted and show deranged activity [78]. Specifically, these T-cells not only are unable to exert effective immune surveillance, but also confer a pro-tumorigenic effect, by accelerating liver damage [78,79]. This unnecessary liver stress may in turn induce premature senescence on hepatocytes, which due to impaired immune surveillance [78], are not effectively cleared and subsequently lead to senescence-related hepatocarcinogenesis.

### 3.4. cGAS-STING Pathway: Linking Stress and Inflammation with Senescence and Cancer

The cyclic GMP–AMP synthase (cGAS)–stimulator of interferon genes (STING) pathway was recently identified as a key factor in liver disorders [80], including HBV infection [81], HCV infection [82], and NAFLD [83]. Activation of the cGAS-STING pathway by endogenous or viral nucleic acids leads to an immune-mediated cytokine release, including the activation of the Type I interferon (IFN-I) pathway [80,84]. While the cGAS-STING seems crucial in viral infection containment and tumor immunosurveillance, it also promotes hepatocyte injury and inflammation, especially in NALFD [80,85]. Importantly, this pathway may be the molecular link between stress disorders and senescence. In a previous study, damaged DNA was associated with cGAS and subsequent deletion of cGAS mitigated senescence [86]. Nevertheless, the authors provided evidence that cGAS may also regulate senescence via unveiled pathways, beyond STING. Glück et al. presented similar, consistent findings [87]. The cGAS-STING pathway is reported to benefit liver tumor immunosurveillance in the short- term, however its long-term activation may promote hepatocarcinogenesis via damaging the tissue and creating an environment of persistent inflammation [88,89,90]. The aforementioned characteristics are reminiscent of the also dual role of senescence, discussed in the Section 2.2 of the present review, further highlighting the link between the cGAS-STING pathway and senescence.

## 4. Senotherapeutics in HCC: A Promising Field

**Figure 2:**
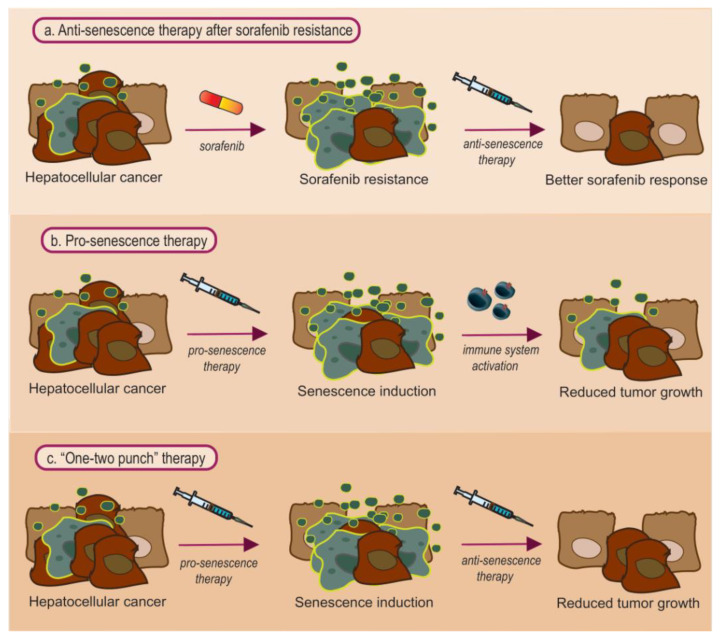
Proposed senotherapeutic strategies: (**a**) anti-senescent agents may be especially useful against sorafenib resistance, a condition associated with senescent cell accumulation. Sorafenib induces the formation of senescent cells, in which it is no longer effective. Thus, a combination of sorafenib with an anti-senescent agent may result in better sorafenib response. (**b**) Pro-senescent agents may have the potential to halt tumor growth via inducing senescence. Thus, malignant cells enter proliferative arrest, and provided that the immune system works, most senescent cells are cleared in an immune-mediated fashion. (**c**) The combined “one-two punch” approach. In this treatment strategy, one starts with a pro-senescence agent, to promote senescence of malignant hepatocytes. As senescent cells may start to accumulate, an anti-senescent agent is administered in the next step, in order to enhance senescent cell clearance. Normal hepatocytes are presented in pale brown, malignant ones in dark brown, whereas senescent ones are presented in light blue with yellow outline.

The role of systemic cancer therapy is either to eliminate the tumor or to arrest its growth. Apoptosis induction usually occurs at the former, whereas the latter may be induced by cell senescence [91]. Currently, the beneficial role of therapy-induced senescence on survival is controversial, while many suggest that this therapeutic mechanism is important [92]. Overall, the idea of senescent cells eventually being cleared by the immune system leads to application of pro-senescence therapy [39,93]. This is also supported by the fact that oncogene-induced senescence is a natural mechanism against tumor growth [17,18]. However, over-accumulation of senescent cells may instead have detrimental effects. Drug-resistant tumors may emerge either through the action of the SASP factors or through the escape of cells from the senescence state and the emergence of drug-resistant cell strains [91]. Therefore, it is proposed that enhancing the elimination of senescent cells may improve therapeutic efficacy and also ameliorate the adverse effects of systemic therapy [94]. Anti-senescence therapeutic agents may be divided into further categories; senolytic, senoptotic, senomorphic, and senostatic drugs [4,95]. Specifically, senolytic drugs aim at enhancing the clearance of senescent cells, senoptotics at inducing apoptosis in senescent cells, and senomorphics at inhibiting factors of the SASP phenotype [4]. On the other hand, senostatics mainly aim at blocking the paracrine propagation of senescence to adjacent cells [95]. Depending on the different therapeutic aims (i.e., to prevent HCC, cure HCC, or overcome sorafenib resistance), different senescence modulating agents may be appropriate. Figure 2 presents possible senescent modulating therapies in HCC, which are analysed below.

### 4.1. Anti-Senescence Therapy: Focus on Sorafenib Resistance

Primary and secondary sorafenib resistance seem to have similar molecular pathways connected to cellular senescence even though the connection does not seem clear [96,97]. Initially, Niu et al. first established a positive correlation between cellular senescence and therapy resistance [96]. The authors observed a higher proportion of senescent cells in the sorafenib resistant cell line compared to sorafenib sensitive cell line [96]. In further experiments, the researches proved that the induction of cellular senescence (observed by SA-β-gal activity, p16 upregulation and IL-6 overexpression) lead to acquisition of therapy resistance [96]. Furthermore, the activation of the AKT pathway was observed in the resistant cells, which however was halted when siRNA knockdown of p16 or antibody neutralization of interleukin 6 (IL-6) was attempted in these cells to by-pass cellular senescence [96]. IL-6 was chosen because it is a dominant cytokine of the SASP phenotype leading to paracrine regulation and promotion of therapy resistance [26,96]. Subsequently, inhibition of either of these senescence-associated molecules resulted in reversion of sorafenib resistance. Importantly, improved sorafenib responsiveness was reported after inhibition of p16 or IL-6 even in primary sensitive cell lines [96].

Activation of the AKT pathway promoting cell survival and proliferation was also observed by Leung et al. when studying the upregulation of Src-homology 2 domain–containing phosphatase 2 (SHP2) in resistant cell lines of HCC and patient derived tumor xenografts [97]. Still, blockage of SHP2 with SHP099, a novel selective orally available SHP2 inhibitor, promoted the re-sensitization of HCC cells leading to apoptosis and cellular senescence. Strikingly, it seems that cellular senescence, induced by SHP099, may have contributed to the observed re-sensitization effect [97].

Understanding mechanisms that contribute to this cell alterations is critical for the successful therapy of HCC [14]. The aforementioned studies on the role of senescence in sorafenib resistance seem somewhat conflicting, but upon considering the context-dependent role of cellular senescence, the differences in the factors of the SASP phenotype may be important. Nevertheless, both reports strongly suggest a role of cellular senescence in sorafenib resistance. Considering that sorafenib and other TKIs constitute first- and second-line systemic treatment in patients with advanced HCC, future studies should focus on elucidating the interconnection of cell senescence with the resistance to these drugs.

Beyond sorafenib resistance, recent studies exhibited promising results regarding the role of anti-senescent agents in HCC treatment. Baar et al. designed FOXO4-DRI, a peptide that aimed to interfere with FOXO4, a transcription factor that promotes the viability of senescent cells. FOXO4-DRI was shown to reduce the viability of Doxorubicin-induced senescent liver cells and protect against liver chemotoxicity [98]. Although FOXO4-DRI was not directly tested against cancer, the authors concluded that it may serve as a promising peptide against cancer progression. Another promising agent is ARV825, a BET (bromodomain and extra-terminal domain) degrader [99]. Wakita et al. observed that it possesses the ability to eliminate senescent cells and protect against HCC occurrence in an obesity-induced mouse model of HCC [100]. Importantly, it was also shown to target chemotherapy-induced senescent cells, and subsequently increase the efficacy of chemotherapy, suggesting its potential usage as a combination drug.

Furthermore, direct-acting antiviral agents (DAA) have revolutionized HCV infection treatment, offering sustained virologic response (SVR) in the vast majority of patients [101]. As the numbers of SVR-HCV patients increased, an important question was whether these patients maintain an increased HCC risk. It was revealed that although this risk is reduced, it remains considerably high several years post-SVR, especially among patients with cirrhosis and the elderly [102]. Particularly, it has been reported that HCV-induced epigenetic changes associated with hepatocarcinogenesis persist after viral eradication, particularly in cirrhotic patients [103]. In addition, the accumulated senescent cells (parenchymal and non-parenchymal) in chronic HCV infection are generally considered to be in an irreversible state [15], and may require considerable time for the organism to clear them effectively and revert back to a healthier state. The aforementioned may partially explain the sustained increased HCC risk during the post-SVR period, and SVR-HCV patients may benefit by the administration of anti-senescence therapeutic agents (Figure 1).

### 4.2. Pro-Senescence Therapy

It has been observed that oncogene-induced senescence may occur in hepatocytes which are subsequently cleared by CD4^+^ T-Cells [39]. In instances where tumor suppressor genes are impaired, no barriers exist against uncontrollable cellular proliferation, thus a pro-senescence therapeutic approach may be an effective treatment strategy. For example, DLC-1 (Deleted in Liver Cancer 1) is a tumor suppressor gene found deleted at approximately 50% of HCCs [104]. Its absence promotes the localization of MKL1 and 2 (Megakaryoblastic Leukemia 1 and 2) proteins in the nucleus, which act as co-activators of the SRF (serum response factor) [105]. The SRF seems to be involved in crucial cellular processes involved in cancer occurrence, such as cell growth, proliferation and migration [105,106]. Therefore, in liver cell lines depleted of DLC-1, it has been observed that the knockdown of MKL1 and 2 reduces cellular proliferation [107]. However, and strikingly, reduction in cellular proliferation was achieved via oncogene-induced senescence. This may be a characteristic example where senescence reveals its positive side as an initial barrier against cancer occurrence.

### 4.3. Combined Pro- and Anti- Senescence Therapy: Is It the Best Approach?

It is generally suggested that a combination of pro-senescence therapy to halt tumor growth, with anti-senescence therapy to clear the senescent cells may be an effective senotherapeutic treatment approach [17]. In line with this hypothesis, Wang et al. attempted to first use XL413 (a potent inhibitor of the DNA replication kinase CDC7) to induce senescence in malignant hepatocytes with mutations in TP53. Next, they interestingly identified the antidepressant sertraline as a senolytic agent that exerts its action via suppressing mTOR signalling. The administration of both agents in a combined, “one-two punch”, approach was shown to effectively reduce tumor growth [108].

## 5. Future Perspective

All data indicate that cellular senescence represents a highly complex process. Although we may be still far from fully unraveling its implication in cancer, recent discoveries highlight its critical role in the development and progression of HCC, mainly by modifying the tumor’s microenvironment. Thus, senescence-modulating agents (senotherapeutics) seem promising towards revolutionizing hepatocellular cancer therapeutic strategies and developing new pharmacological approaches. Still caution is required, as the current knowledge on the molecular pathways behind senescence and senolytics is relatively limited and therefore further studies are warranted, keeping in mind the high heterogeneity of HCC. Chronic HBV infection represents a major risk factor for the development of HCC and considering that HBx gene is one of the most frequently mutated genes during HBV chronic infection, exploring the impact of mutated types of HBx on senescence would upgrade our understanding. Furthermore, as the complexity of both HBV and HCV infection leads to multiple immunomodulatory mechanisms in the tumor’s microenvironment, affirming whether this is the reason behind senescence-mediated HCC occurrence could wider our prospective for new interventions. However, considering that the domination of viral hepatitis as a risk factor for HCC is being overcome, research should also focus on NAFLD-mediated HCC. In NAFLD, alterations in the gut microbiota seem to result in the secretion of metabolic factors that induce senescence in hepatic stellate cells and create a permissive tumor microenvironment. Revealing the mechanisms through which senescent hepatic stellate cells communicate with hepatocytes in order to promote carcinogenesis may result in the development of therapeutic agents that interfere with this pathway.

## 6. Conclusions

The review herein aimed to present data on the role of senescence in the development and progression of HCC in three major liver conditions; HBV and HCV infection as well as NAFLD. Recent discoveries in these conditions highlight a critical role of senescence in HCC (Table 1). Furthermore, data suggest that senescence contributes in resistance development during the administration of targeted therapies, such as sorafenib. Although we may still be at a primitive stage in fully unraveling the role of senescence in cancer, it seems that senescence-modulating agents (senotherapeutics) have the potential to revolutionize the way we treat hepatocellular cancer. Thus, focusing our research towards senescence-mediated HCC may provide the missing elements required to identify and even halt precancerous stages of HCC.

## Figures and Tables

**Table 1:** Contribution of senescence in HBV-, HCV- and NAFLD- mediated HCC: Key elements.

Liver Condition	Key Elements
**HBV infection**	***Isoforms of the hepatitis B virus X (HBx) protein*** Different effects on senescence depending on the isoform Promotes factors of the SASP phenotype May enhance telomere shortening, and thus trigger replicative senescence and cirrhosis
**HCV infection**	***Increased presence of senescent T-cells*** Do not effectively clear pre-malignant hepatocytes ***HCV core protein*** May promote the evasion of normal stress-induced senescence, allowing damaged cells to proliferate
**NAFLD**	***Gut microbiota and senescent hepatic stellate cells*** Gut microbiota secrete DCA and LTA, which enter the circulation and promote hepatic stellate cell senescence as well as the expression of factors of the SASP phenotype

DCA = deoxycholic acid; LTA = lipoteichoic acid; HCC = hepatocellular carcinoma; HBV = hepatitis B virus; HCV = hepatitis C virus; NAFLD = non-alcoholic fatty liver disease; SASP = senescence-associated secretory phenotype.

## Data Availability

Not applicable. The current study did not generate a new dataset.
